# Reflective Thinking Skills of Academic Administrators in Higher Education

**DOI:** 10.3389/fpsyg.2022.893517

**Published:** 2022-06-21

**Authors:** Gamze Kasalak, Miray Dağyar, Mehmet Özcan, Etem Yeşilyurt

**Affiliations:** ^1^Department of Educational Sciences, Akdeniz University, Antalya, Turkey; ^2^Department of Educational Sciences, Nevşehir Hacı Bektaş Veli University, Nevşehir, Turkey

**Keywords:** reflective thinking, reflective implementations, higher education institution, academic administrators, reflective thinking skills

## Abstract

The aim of this study is to determine the reflective thinking skills and reflective implementations of academic administrators in higher education and to reveal the importance of reflective thinking for higher education. The research was carried out in a holistic single case design as a case study which is known as one of the qualitative research methods. The study group of the research consists of 12 faculty members who carry out administrative duties (dean and assistant dean) in ten different faculties of a public university. The data collected through a semi-structured individual interview form were analyzed by content analysis technique. According to the results obtained from the research, it has been determined that academic administrators acquire their administerial skills mostly based on their experiences, their own competences, and their tendencies to evaluate their actions individually are high, and they also consider internal and external evaluations. Reflective thinking areas related to faculty management and individual development of academic administrators were also determined, and it was concluded that they questioned faculty management mostly in terms of education and student services, and their individual development in terms of problem solving and communication skills. In addition, in the study, the characteristics of reflective academic administrators and the contributions of reflective thinking were determined based on the views of academic administrators. Finally, metaphors describing the reflective-thinking academic administrator were included, and it was found that academic administrators have a difficult job based on their management duties, they should approach everyone equally and be in the role of administrators, and that the administerial task is temporary.

## Introduction

### Problem Statement

In the literature, there are studies which focus on reflective thinking abilities of teachers (Pazhoman and Sarkhosh, 2019; Zahid and Khanam, 2019; Özdemir and Oruç, 2020; Tunç and Kıncal, 2021), preservice teachers (Dervent, 2015; Tican and Taşpınar, 2015; Choy et al., 2017; Adatepe and Kul, 2018; Elmalı and Kıyıcı, 2018; Öztürk, 2018), and students (Kember et al., 2000; Ersözlü and Arslan, 2009; Qamar et al., 2017; Kheirzadeh and Sistani, 2018). However, it has been determined that there is a limited number of studies on the reflective thinking skills of school administrators (Ghanizadeh, 2017; Lee et al., 2017; Altan, 2018; Henriksen and Aas, 2021). Also, the studies hat focus on administrative staff working at universities mostly limited to investigate the leadership skills of administrative staff (Bellibaş et al., 2016; Sharma et al., 2016; Uslu, 2016; Karaaslan and Akın, 2019). Reflective practice is important for the development of all professionals. Although we provide our learning with experience, more experience does not mean more learning. For this reason, it seems that the ability of managers to turn their experiences into learning is the skill they need most, which is the basic principle of reflective thinking (Dalgıç, 2011). A reflective school administrator becomes a good role model for the teachers and students around him. The school administrator, who makes learning and questioning his routine, reflects this feature on all practices in the school and indirectly acts as a catalyst for teachers and students to be trained as reflective practitioners (Dana, 2009).

The rapidly changing needs of the 21st century is directly reflected in higher education and academic administrators are expected to be able to respond to rapidly changing needs. Therefore, in this process, the need for reflective thinking practices, in which academic administrators transform their experiences into learning and improve their practices by constantly questioning, is felt more than ever in order to accelerate their adaptation to new conditions. In this respect the current study was developed based on the views of academic administrators at the higher education level, has great potential to contribute scientific knowledge. Furthermore, it can be said that the research will encourage other researchers working in the field of management in higher education to conduct similar studies and thus contribute to the spread of reflective thinking skills. It is expected that the results of the current research will shed light on the effectiveness of reflective thinking skills and will give new ideas to researchers.

The aim of this research is to describe the reflective thinking skills of academic administrators who work as deans or vice deans in higher education, based on the experiences and opinions of university administrators. The following questions were directed to be answered:

1. What are the opinions of academic administrators on the “ways of learning management”?
2. What are the opinions of academic administrators on “methods used to evaluate managerial competencies and actions”?
3. What are the opinions of academic administrators on “people they interact with in the reflective thinking process”?
4. What are the opinions of academic administrators on “reflective thinking areas”?
5. What are the opinions of academic administrators on “characteristics of administrators who possess reflective thinking skills”?
6. What are the opinions of academic administrators “on the contribution of reflective thinking to administrators”?
7. What are the academic administrators’ opinions on the “metaphor developed for the reflective administrators”?

## Literature Review

Reflective thinking is the individual’s questioning and criticism about their past and present experiences, self-evaluation and thinking about what they can do to solve the problems that arise because of this evaluation (Özdemir, 2018). Reflective thinking consisting of self-questioning which can be achieved by the individuals who are resourceful, free from stereotypes and capable of thinking critically while challenging oneself with the following questions: ‘What have I done? What am I doing? What do my actions state?’ (Roskos et al., 2001). In this respect, reflective thinking is a meaning-making process that allows a learner to move from one experience to the next with a better knowledge of how it relates to and connects to other practices and concepts (Rani, 2022). Reflective thinking is thinking deeply about learning/teaching and thinking process, self-evaluation and working on solutions (Elmalı and Kıyıcı, 2018). Reflective thinking helps arise the strength of an individual while searching a solution for the problems (Kholid et al., 2020).

Dewey (1933) stated for the first time the phenomenon of reflective thinking and his ideas on the concept of teaching in his book “How We Think” as follows: “Everything changes, nothing stays the same. Times change, people change, everything changes… and if you are not a reflective teacher,” “You can’t change with them. And if you don’t change you won’t be effective. You have to be ready and willing to adapt to these changes.” Dewey explains the qualities that an individual must have for reflection to occur as open-mindedness, full willingness and responsibility (Kotzee, 2018): Open-mindedness is the ability to look at the problem from different and new perspectives. Being open-minded requires being an active listener, being ready to listen to others, and understanding that their beliefs may be wrong. Full willingness occurs when a concept is fully involved. It integrates with experiencing many ideas and thoughts. Responsibility is taking the consequences of one’s actions. It is the need to know the reason, to seek the meaning in what is learned. Dewey explained the five stages of reflective thinking as follows (Priest, 2021): (1) Suggestions in which the mind leaps toward a possible solution; (2) In the context of a problem that needs to be solved or an answer to be found perception of difficulty or complexity; (3) Initiating and directing observations for the collection of realistic materials and other a proposal as a leading idea or hypothesis to initiate operations to use; (4) Mental elaboration of an idea or assumption; (5) Testing of hypothesis through explicit or imaginary action.

The general purpose of reflective thinking is to understand a situation, an event or a piece of information and better solve the existing problem. Reflective thinking consists of claims, problems, hypotheses, reasoning, and testing. In order for the student to think fully reflectively, he or she should follow the following stages in the order given. These stages are listed by Dewey as follows (Bustami et al., 2018): (1) Experience, (2) Self-understanding of experience, (3) Naming problems or questions that exist outside of the experience. (4) Developing positive explanations for posed problems and questions, (5) Branching out explanations within fully developed hypotheses. (6) Testing selected hypotheses.

Individual who are able to think reflectively for self evaluating and evolving tend to adopt reflective implementations. In this respect, reflective teaching is a good method for both preservice and in-service teachers’ professional growth (Mathew et al., 2017). Teachers with a high level of preparedness for learning and reflective thinking are successful in controlling the teaching and learning process, successfully utilizing knowledge sources, sustaining motivation, having good problem-solving abilities, and serving as a role model for students (Gencel and Saracaloglu, 2018). Therefore, reflective thinking can be described as the basis of enhancing impementation, developing skills and organizational learning (Dohn, 2011). Schön argues that professionals apply two kinds of reflection in making sense of and developing their actions. These; reflection during action and reflection after action. Dimensions of projection are metacognitive processes that have an orientation to guide future actions. Stating that the most striking examples of reflection occur during the action, Schön states that reflecting after the action will enable learning (Edwards, 2017). Reflection in action is the individual’s thinking in detail about the action he is performing. In reflection in action, the individual unconsciously uses his knowledge during the action and reflects to better understand the action. With the continuity of reflective thinking, he uses the data he has obtained about his own actions in shaping the actions he will take in the future. In reflection during the action, it is necessary to think and evaluate in detail the student-teacher interaction in the teaching process. Reflection after the action is the individual’s re-evaluation of their practices and reflection on their practices (Ellis, 2020).

Reflective trainers are expected to have the characteristics of self-efficacy, flexibility, social responsibility, and metacognition (Colton and Sparks-Langer, 1993): Without a sense of self-efficacy, educators are not motivated to evaluate their own practices and seek deeper meaning in them. Flexibility is important as reflection requires looking at the world from different perspectives. Educators with a sense of social responsibility engage in and engage in behaviors that are beneficial to their students, schools, and communities. Reflective teachers have a sense of improving their teaching skills and dispositions to teach better. Metacognition means being aware of one’s own thoughts and decisions.

## Materials and Methods

### Research Pattern

The current study was carried out in a holistic single case design, which is a qualitative research type of case study. In a holistic single case study, a single unit of analysis (an individual, an institution, a group, an environment, a problem) is studied. During the case study, the interaction between the factors and the certains situations (environment, individuals, events, problems, processes, etc.). Therefore, in this research, which was carried out on the basis of the interpretive paradigm, a phenomenon was examined in depth and contextual explanation was made on the basis of the questions of “how” and “why.” As a result, in this research, it is aimed to describe the reflective thinking skills and practices of academic administrators in higher education based on the experiences and opinions of the faculty members who work in higher education administration.

### Study Group

The study group of the research consists of 12 faculty members who carry out administrative duties (dean and assistant dean) in 10 different faculties of a public university in the 2019–2020 academic year. The study group of the research was determined by the criterion sampling technique, which is one of the purposive sampling methods. The purpose of selecting participants in qualitative research is to reach the most appropriate people who will deliberately lead to the best understanding of the research problem handled in the research (Cresswell, 2017). While determining the study group, four criteria were taken into consideration. These criteria are (a) The participants are actively continuing their administrative duties in a higher education institution (b) Working in faculties representing different disciplines (Health, Social and Science fields), (c) Volunteering to participate in the research. The study group of the research is given in Table 1.

**TABLE 1:** Personal information regarding the study group.

Participant	Gender	Faculty	Academic field	Academic title	Administrative title	Total years of professional service	Years of service in the relevant administrative title
FHS1	Female	Nursing School	Health	Prof.Dr.	Dean	3	8
MS2	Male	Engineering School	Science	Prof.Dr.	Dean	25	4
MS3	Male	Faculty of Science	Science	Prof.Dr.	Dean	42	1
MSoS4	Male	Faculty of Education	Social	Prof.Dr.	Dean	27	3
MSoS5	Male	Law School	Social	Prof.Dr.	Dean	24	4
MSoS6	Male	Law School	Social	Ass. Prof	Vice Dean	15	5
FSoS7	Female	Faculty of Economics and Administrative Science	Social	Prof.Dr.	Vice Dean	26	4
FS8	Female	Faculty of Architecture	Science	Ass. Prof	Vice Dean	9	3
MS9	Male	Faculty of Dentistry	Health	Prof.Dr.	Dean	18	4
FS10	Female	Engineering School	Science	Assoc. Prof.	Vice Dean	2	3
MSoS11	Male	Faculty of Theology	Social	Ass. Prof	Vice Dean	27	1
MS12	Male	Faculty of Architecture	Science	Prof.Dr.	Dean	11	1

Since the research aims to examine the views of academic administrators at university level on reflective thinking skills and practices, it is aimed to represent views from different disciplines in an institutional integrity. For this purpose, academic administrators whose ethics committee permissions were obtained from 10 different faculties located in the central campus of a state university were selected as the study group. Distribution of the participants, four of whom were women and eight men, according to their fields of expertise: social sciences (*f* = 5), natural sciences (*f* = 5) and health sciences (*f* = 2). As academic titles, eight are professors, one associate professor and three assistant professors. As directors, seven are deans and five are vice deans. The total years of professional service of the participants were between 9 and 42; The years of service in the relevant administerial title vary between 1 and 8 years.

### Data Collecting Instrument

In this study, data were collected through a semi-structured individual interview form. Individual interview form consists of three parts: (i) explanation, (ii) personal information form and (iii) academic administrator reflective thinking skills. In the explanation part of the form, information regarding to the purpose, method, and process of the research, what is expected from the participant, voluntary participation in the research and the permission for voice recording were provided. In the personal information part of the form, there were seven questions directed for collecting the identity information of the participants (see Table 1). Academic administrators reflective thinking skills consist of individual interview form, literature review (Dalgıç, 2011; Özdemir, 2018), expert opinions, and eight basic questions and probe questions prepared by the researcher for the purposes of the research based on the pilot application. The initial draft of personal interview form was submitted to the expert opinion of two lecturers who are experts in the field and experienced in the qualitative research area. Nonetheless, the first trail interview has been conducted with a faculty member who is an experienced administrator staff at a high education institution.

The final format of the interview form has been established in the light of the experts’ feedbacks and results of the trial interview. In the interview form, it is aimed to determine the opinions of the academic leaders’ on the following issues: “methods to learn administration skills,” “methods used to evaluate administrative competencies and actions,” “people whom to be interacted with in the reflective thinking process,” “reflective thinking areas,” “academic administrators characteristics possessing reflective thinking skills,” “contributions of reflective thinking to academic administrators,” and “metaphor developed for reflective thinking academic administrators.” 11 out of 12 participants agreed to make individual interviews in the present of a voice recorder, while one participant preferred interviewer would compose the interview in written details. Each interview lasted between 25 and 50 min in the participants’ offices, and all interviews were completed by the researcher in two months.

### Validity and Reliability

In order to ensure the external validity (transferability) of the research, the date and the interview questions were prepared in the format to be used during the interview in the personal interview form. The process of the research, the recording of the interviews and the reporting of the results were explained to the participants in detail. While developing the interview form, earlier studies done by fellow researchers which is directly related to the purpose of the current research, was looked through and examined to ensure the internal validity (credibility) of the research. Furthermore, the theoretical foundations of reflective thinking skills examined ensure that the questions in the interview form had the content and intelligibility suitable for the purpose of the research. The comprehensibility and intelligibleness of the interview questions’ appropriateness for the purpose of research were evaluated through conducting a pilot interview with a faculty member who possess administrative experience, while long-term interaction method was implemented. In this manner, an atmosphere of trust between the researcher and the participants were established to ensure collected data was constructive (Yıldırım and Şimşek, 2013). In order to ensure internal reliability (consistency) in the research, a roadmap was created that includes criteria and 82 thematic frameworks to ensure harmony and unity in the analysis of data. For this purpose, two educational science experts, who have scientific research experience in the field of qualitative research, and two participants, corresponding to 12.5 percent of the total participants, were asked to code the data according to the determined roadmap. In order to determine the consistency between the codes of the experts, reliability analysis was performed in the SPSS 13th package program and the Kohen Kappa consistency coefficient was calculated as 0.87 (*p* < 0.001). The fact that the Kappa coefficient is between 0.81 and 1 and that the coding made by the experts independently of each other are compatible, indicates “consistency of excellence” (Landis and Koch, 1977). In the context of external reliability (confirmation examination) of qualitative research, it has been ensured that the research process is clearly revealed, raw data is archived and open to audit if deemed necessary (Yıldırım and Şimşek, 2013). For this, detailed explanations about the research process were presented to the evaluation of a faculty member working in the field of educational sciences and a confirmation examination was carried out.

### Data Analysis

Content analysis technique, which is defined as “a systematic and repeatable technique in which some words of a text are summarized with smaller content categories with coding based on certain rules” (Büyüköztürk et al., 2018), was used in the analysis of the data. The data were coded in line with the expressions and words representing the basic views in line with the purposes of the research. Themes were formed by bringing together the data coded in relation to each other (Glesne, 2013; Cresswell, 2017). NVivo 1st package program was used for coding and theme creation. Frequency (f) and percentage (%) were also used in the analysis of the data; while direct quotations were included for conveying the opinions of the participants. In order to keep the information of participants confidential, coding was carried out using various symbols (for the participants who were academic administrators). The first character used in the coding shows the gender of the participants (F = Female, M = Male), the second character shows their academic fields (SoS = Social Sciences, S = Sciences, HS = Health Sciences), the third character shows the sequence number. For example, FHS1 is the number one female academic administrators in the field of health sciences; MS2, on the other hand, represents the number two male academic administrator who is an administrator in the field of science. Ethical rules were taken into consideration during the interviews.

## Results

### Methods Academic Administrators Learn About Administration

The “methods of learning administration” of academic administrators are expressed with eight different codes and the relevant codes.

The methods of learning administration of academic administrators are defined with a total of eight codes: Learning from administrative experiences (*f* = 1, 23.8%), through practicing and experiencing (*f* = 1, 23.8%), administrators and colleagues (*f* = 9, 21.4%), other work related experiences (*f* = 5, 11.9%), administrative personnel (*f* = 3, 7.14%), written and visual resources (*f* = 3, 7.14%), state administrators (*f* = 1, 2.38%) and family members (*f* = 1, 2.38%). The opinions of a participant who emphasized the importance on learning academic administrator from family members, administrative personnel, administrators and colleagues and by practicing and experiencing, are as follows: “…*I received a lot of support from the dean, vice deans and our faculty secretary*… *Since my wife is an administrator, she consults and helps me by sharing her experience. I try to learn by practicing and experiencing.*” (FS8). Another participant’s (MS3) opinion on learning by taking state administrators as an example is “*I take Atatürk as an example. I think how Atatürk would have done when a problem arose. I look at everything in a democratic way, I have never given up being a democrat, I will never give up on that. If you look at problems in a humane democratic way, the best solution emerges on its own.”*

### Methods to Monitor and Evaluate Academic Administrators’ Competences and Actions

The opinions of academic administrators on “the ways to monitor and evaluate their competences and actions” are expressed with the sub-themes of “individual,” “internal” and “external” reflection.

The views of FS10, who stated that they make individual evaluations (*f* = 6) while evaluating the strengths and weaknesses of academic administrators, are as follows:

*“. am I good enough for this, do I have an administrative infrastructure or not? Because when you are an academician sitting in your chair in your office, sometimes you can’t wrap your head around about what the processes are that you can’t know and why some of the disruptions happen.*…. *I had hesitations about administration duty, but then I accepted it, thinking that this will be a life experience for me, also I was curious, and I thought I’d get into the business a little bit and see for myself, I realized that it was really very difficult, but*…

Academic administrators use both “formal” and “informal” evaluation methods together in internal reflection. Academic administrators within the institution find the opportunity to monitor and evaluate their own competencies and actions by interacting with “academics” (*f*_*formal*_ = 5; *f*_*informal*_ = 4), “students” (*f*_*formal*_ = 4; *f*_*informal*_ = 4), “administrative personnel” (*f*_*formal*_ = 4; *f*_*informal*_ = 4) and “administrators” (*f*_*formal*_ = 5; *f*_*informal*_ = 2). MS2, who states that they evaluate their competencies by making performance-based quality evaluations with the fellow academicians during the official reflection process, expresses their views as “The faculty members answer questionnaires before each faculty academic board. Our 1-year satisfaction rate is 76%.” MS2 also stated that that informal feedback was provided by fellow academicians for the following questions: “What would you like to fix in me, positively or negatively, what did I do wrong or how should I have done it?”.

A participant, who was in the process of reflecting with the students both formally and informally, said, “I asked students who are attending the classes at our faculty to provide feedback in the context of the Bologna process. Nonetheless, I interacted with social media to give feedback to the students.” (MS12). MSoS4 was another interviewee who conducted a reflective process with students via faculty representatives, in addition to the reflective process done with administrative personnel through social gatherings said that “Demands and expectations can be formed through the faculty secretary… Also, we get together in social environments from time to time. e.g., We had a breakfast meeting with the administrative personnel during which there was opportunity to share thoughts on administrative problems in a conversational atmosphere.”

Academic administrators interact with a variety of people in external reflection while monitoring and evaluating their competencies and actions which are listed as following: external management (*f* = 8), friend (*f* = 4), media/social media (*f* = 4), industry (*f* = 3), legal expert (*f* = 3), family (*f* = 2) and personal development specialist (*f* = 1). Academic administrators make informal assessments with these individuals during the external reflection process. While one participant (FS10) stated that s/he interacted with the former department chair and an academician who was once his/her Ph.D. committee chair, another participant (MSoS4) said, “We have interaction with the board of deans established by universities.” Emphasizing the fact that that there is an active interaction with people who either were or have been appointed as an administrator outside the institution. The opinion of FSoS7, who had done an unofficial reflecting process via social media and WhatsApp groups, is given below:

“*I use all social media channels such as Twitter, Facebook, and Instagram to evaluate our administerial skills. In addition, we also have WhatsApp groups for the flow of information between faculty members. We use social media channels as a management tool, as we hold our post-pandemic meetings and evaluations in the form of expressing opinions and making decisions.”*

The opinion of FS10, who is in the reflective thinking process with her/his family, is as follows:

“… *my father served as an administrator for many years as the assistant principal at National Education Ministry’s Antalya office. Sometimes, in situations that really have bothered me, I have asked him what he would do if he were me, and I try to figure out a way to solve the problems by combining the answers he gives with the answers I have gained during my term in the office*…*.”*

### Reflective Thinking Areas of Academic Administrators

The “reflective thinking areas” of academic administrators are expressed with sub-themes related to individual development and faculty administration, and the relevant codes are given in Table 2.

**TABLE 2:** Reflective thinking areas of academic administrators.

Faculty administration related fields	f	Individual development areas	f
Education and student services	8	Communication	3
Higher education legislation	5	Problem solving	2
Faculty resources management	4	Sociology content philosophy	1
Personnel services	3	Knowledge of legal rights and freedoms	1
Psychological aspect of the faculty	2	numerical field skills	1
Technology	2		

When Table 2 is examined, reflective thinking areas related to faculty administration are coded as following: education-training and student services (*f* = 8), higher education legislation (*f* = 5), faculty resources management (*f* = 4), personnel services (*f* = 3), psychological aspect of the faculty (*f* = 2) and technology (*f* = 2). Additionally, personal development areas of the academic administrators’ codes are communication (*f* = 3), problem solving (*f* = 2), sociology content philosophy (*f* = 1), knowledge legal rights and freedoms (*f* = 1) and numerical field skills (*f* = 1).

MSoS11, emphasizing the fields related to education-training and student services, higher education legislation, management of resources and technology, expressed her/his views as follows:

“…*it is necessary to read the higher education legislation in detail, such as the creation of course syllabuses, the registration of students. Since it is constantly changing, it is necessary to follow the changes and learn about the updates*… *I would like to learn what needs to be done for effective use of resources*… *I would like to attend all relevant meetings to use the system where we carry out distance education applications and information management processes more effectively.”*

MSoS4 emphasized human relations and democratic working environment related to the psychological aspect of the faculty which is expressed in the following words:

*“There is a legislative part of this administration, and there is a part that touches people, for example, I think that people are very valuable. I would like to have a working environment where everyone feels valued. A working environment where no one is marginalized*… *If we need education, I think it can be democratization of the working environment or creating an environment where people are important and valuable.”*

The most important issues related to individual development areas are communication and problem solving, and a participant (FHS1) on this subject said, *“Academy administrators need to develop oneself and receive training on interpersonal relations and problem solving, both for students and academic staff. Higher education is a very dynamic process, not a static system, but a living system. There are constant innovations and changes. You have to keep up with it”.*

MSoS5’s opinions on legal rights and freedoms and Sociology content philosophy are as follows:

*“Academic administrators must have training in the field of law since independent of what department we are appointed as an administrator we practice the law. People who expect a solution from us also want the solution we put forward to be in accordance with the law. We are also aware of the fact that we must abide to the fact that the work we do is following the law. Therefore, we need to have a basic knowledge of law. In other words, we need to know what fundamental rights and freedoms are.*

*Administrators do not have the luxury of not meeting with people, getting angry with people, or feeling resentful to people. The seat they are filling in is at an equal distance to everyone, and if it is a problem arising from the institution they serve for, regulating it is one of their obligations. The one cannot ignore the problems, or remain unresponsive, for these law and sociology are required. Therefore, they need to do research and read the basic information about sociology.”*

### Characteristics of Reflective Thinking Academic Administrators

The competencies that a reflective academic administrators should have, were expressed by the participants as follows: Unbiased, objective, fair, problem-solving, constructive, strong in human relations and communication (listener, empathetic, cooperative, devoted, responsible, tolerant, honest, reliable, respectful, supportive, making others feel valued), open-minded (open to modernization, learning and adjustments), persistent and purposeful thinking, accessible. On being open-minded, FS10 said, *“If the administrator has never improved oneself, if the one is not open to any innovations, or to any opinions, then there is no point in being in that position. The person called an administrator must be fair, open minded and foresighted, must be able to develop oneself, must be open to modernizations*…” Furthermore, FHS1 said that *“You must keep up with constant modernizations and* adjustments, *you need to receive continuous training when you encounter new developments. e.g; Distance Learning. “A participant on human relations and communication stated, “As an administrator, you need to have the ability to listen well, you need to be able to think about delicacies, you need to empathize*…” (MSoS4), while expressing their opinions; another participant’s opinions are as follows (MS2):

“*The one should know the legislation and listen to the stakeholders, meet with the students, the teacher, the department heads and senior managers at certain intervals*… *One of the very important issues is: there must be a good team, there must be effective working commissions*… *If there is no team that is compatible with each other, administrative processes are blocked and forced. This is a harmonious and devoted team established by vice deans, department heads and vice department heads. The most critical issue of the administrator is the team. The mistake made by someone very important to your team is your fault.”*

### Contribution of Reflective Thinking to Academic Administrators

The contributions of reflective thinking to academic administrators are expressed in five different codes as communication, decision making, coordination, organization and planning and development of evaluation skills.

The contributions of reflective thinking to academic administrators are to develop communication (*f* = 8), decision-making (*f* = 7), coordination (*f* = 6), organization and planning (*f* = 4) and evaluation (*f* = 3) skills which are defined by a total of five different codes. The opinions of the participants who shared their contributions to communication skills are as follows*: “I learned to be calm*…. *Administration position has increased my problem-solving speed*” (FHS1), *“I learned where, when and what to talk about* (MSoS6).” *“You get to know the faculty. I had the opportunity to observe what kind of people there are, what characteristics they have, what expectations they have.*… *The biggest motivation of the administration is to find solutions, to be able to solve people’s problems*… *this is the biggest achievement for me.”* (MSoS5).

Regarding decision making, MS12 said, *“I learned to make quick decisions and be practical in a crisis environment.”* MS2 *“I started to decide more quickly how to do the duty and how to do it.”* And, finally, MSoS5 expressed their opinions as, *“No matter what position you are in, you are calculating the applicability of the decisions you make and what kind of the benefits will be stemmed by the action that you take about the people even if those decisions and actions will not be directly associated to the institution you work at.”*

Regarding the coordination code, FHS1 says, *“You learn when to set corporate goals and when to work like an orchestra conductor, and when to rest, that is, to be like a great conductor.”* Regarding the organization and planning code, MSoS5 said, *“Most importantly, you gain the ability to manage meetings. A newly appointed administrator can complete a job in 2 h, while an experienced administrator can complete it in maybe 45 min.”* Lastly, MS9 said that *“During the establishment of this faculty, I had to act as if I were the power plant, carpenter, architect, and interior architect etc.*…. *When I look back, I am happy because of what I had dealt with during the process. you are improving yourself, you are thinking practically*.” Regarding the evaluation code, MSoS11 said, *“First of all, it is necessary to follow up, but any step we take needs to be followed and evaluated”.*

### Metaphors Developed for Academic Administrators

The metaphors developed for the reflective-thinking academic administrator are expressed in four different sub-themes: profession and community, nature, space, and object.

Regarding the profession and community subtheme, military service (*f* = 1), football coach (*f* = 1), conductor (*f* = 1) and orchestra (*f* = 1); regarding the subtheme of nature, thorn (*f* = 1), ramp (*f* = 1), mountain climbing (*f* = 1), and regarding to the environment, place of worship (*f* = 1) and pyramid (*f* = 1);, balloon (*f* = 1) and flag (*f* = 1) metaphors were developed. Regarding the metaphor of military service in the sub-theme of profession and community, FSoS7 said, “*We have a great public service duty that needs to be done as soldiers do*…” and regarding the metaphor of “Orchestra”, FHS1 said *“There are too many instruments in higher education. Relations with students, academicians, administrative personnel, senior management, society must be handled all well and the melody should sound nice. It makes a beautiful melody when they all harmonize together, not alone.”* The opinion of a participant (MSoS6) is quoted as it is in the below regarding the metaphor of “thorn” which is in nature sub-theme:

*“I think, an academic should stay away from being an administrator. It’s something that I don’t think anyone should do. You make enemies, not friends. Academic studies are secondary. It’s a no-non-sense position. If you have different personality traits, you can turn this into a plus. It varies from person to person, but like I said, it’s something everyone should stay away from. Then we can call it a thorn.”*

A participant’s view on the metaphor of “climbing the mountain” may also be important in clarifying the challenges of academic administrator:

*“It has both difficulty and beauty. You are both active and tired. It’s like carrying the universe on your back. I mean, you go out and you see very beautiful sceneries. However, in one place, the wind can blow very hard, you can slip and fall. Stones and avalanches are falling on you, but you have to stand ground. Whatever happens. You know, you have to keep your feet and hands very strong and make your decision very well, you have to take your steps firmly. I think it’s like climbing a mountain.”* (MS9)

A participant (MSoS11) on the metaphor of a place of worship developed in the sub-theme of environment said, *“I relate it to a place of worship. Anyone can enter and leave the place of worship without question.”* Regarding the balloon metaphor developed in the sub-theme of the object, FS10 said, *“Like a balloon, you start inflating, there is no problem when inflating; you can anticipate when the balloon will burst. As the balloon gets bigger, you like it very much. But then you say: Okay, it’s time to stop inflating. I am at that point right now.”* Regarding the flag metaphor, FS8 shares the following: *“This is a task that needs to be done, I mean, this is a job, a service that needs to be done for the state and the nation. This is how I see it, now I’m doing it, then someone else will do it. So, it’s like a flag circulating from hand to hand. “*

## Conclusion

In the study, the methods of acquiring management skills of academic administrators, the styles of evaluating their abilities and actions, the way they question faculty management and their individual development, the characteristics of reflective academic administrators and the contributions of reflective thinking to administrative staff were investigated. Furthermore, metaphors related to reflective thinking academic administrators were examined.

It has been determined that academic administrators get promoted to the administrative positions based on their previous administrative experiences. In other words, academic administrators do not need to have a specific education for administrative position, and task assignments are usually given in reference to previous administrative experiences that has been gained at lower ranked administrative office duties. According to Pocklington and Weidling (1996), it is not possible to fully prepare a school administrator candidate for the profession in advance of taking the office. The tendency of academic administrators to be administrator staff based on their experiences reveals the importance of reflective thinking skills, which can enable them to turn to the right direction by questioning their experiences. Dalgıç (2011) also stated in the relevant study that school administrators in Turkey are focused on learning while doing their duties and learning from their experiences, therefore it is important for them to have reflective thinking skills to turn experience into learning. As a matter of fact, for an acquired experience to turn into learning, a meaning must be attached to the experience by the administration, and this is only possible with reflective thinking (Raelin and Coghlan, 2006). In addition, according to the opinions of academic administrators, it was determined that the tendency to learn administrating skills from visual or written sources was low. However, according to the literature, it has been determined that academic administrators have training needs for professionalization in administration (Dimici et al., 2016) and there are orientation demands especially for leadership training and administrative duties (Bellibaş et al., 2016).

It has been determined that internal and external evaluations are made in addition to individual evaluation that is among the ways to monitor and evaluate the competencies and actions of academic administrators. Besides, it has been determined that administrators evaluate their strengths and weaknesses mostly by questioning oneself. In this context, reflective thinking had better be experienced primarily at the individual level in the event of organizational change and development is purposed. A manager should systematically question their own managerial actions and practices and make the necessary changes as a result (Dana, 2009). Administrators should first question their own competencies, make changes in their actions, and then expect a change in the education system (Osterman and Kottkamp, 2004). In addition, the evaluations of academicians, students, administrative personnel, and other administrators in the institution are considered important for managers. In the internal evaluations, it was stated that the feedback of the academicians and students, formally or informally, on the competencies and actions of the administrators is important. The reason for this can be shown as the fact that the masses directly affected by the decisions taken by academic administrators at universities are students and academics. Since students and academics are at the center of their services, academic administrators are expected to question their competencies and take their actions into account, considering the views of academics and students. As a matter of fact, reflective thinking allows educators to see oneself through the eyes of students and makes teachers and administrators feel more responsible for questioning their own competencies. In addition, reflective practice allows for the accompaniment of teacher training that, rather than beginning with theoretical knowledge, begins with personal and professional experience, promoting the improvement of their classroom task (Cappelletti and Sajon, 2022). Managers who think reflectively do not want to establish authority over other members of the organization, because this creates a culture of consensus at the institution and prevents the development of different perspectives on events. It is impossible for an administrator who has created this environment to change and develop their actions and practices according to internal evaluations (Hargreaves, 2003).

In terms of external evaluation, it has been determined that managers are mostly affected by external management, colleagues, and media/social media. In other words, as external administration, it is expected that administrators had better consider a senior institution administration and their close circle for evaluation. In addition, administrators are aware that the media is an important factor in questioning their competencies and actions in today’s technology era. In her study, Dalgıç (2011) recommends that school administrators take part in communication networks that bring together different administrators from outside the institution to make their learning continuous. It is also pointed out that the communication network activities of school administrators (annual meeting, seminar, conference, etc.) should also be supported by online forums (Dalgıç, 2011). In today’s conditions, administrators are expected to include certain social networks in their communication networks and share with internal and external academicians, administrators, and students to find solutions to current problems.

Academic administrators’ reflective thinking areas regarding faculty administration are mostly stated as education-training and student services and higher education legislation. The reflective thinking areas of administrators on individual development are communication, problem solving, sociology content knowledge, legal rights and freedoms knowledge and numerical skills. The reason of principals’ questioning faculty administration is mostly education-training, student services and higher education legislation as these are the primary duties of administrators. It is recommended that school administrators attend classes for a few hours a week so that they do not fall behind in educational practices (Hargreaves, 2003). The reason for this may be that the administrator oneself takes part in the education process and practically questions the effects of administrative decisions and actions on the school culture and the services provided to the students. According to Pocklington and Weidling (1996), administrators should be directed to in-service training in terms of administrative knowledge. This may happen due to the fact that administrators question oneself and contemplate reflectively, focusing on communication and problem-solving skills primarily, and believe that this will more efficiently help them coping with responsibilities related to the administration of the faculty.

Reflective thinking enables administrators to look at an event from different perspectives, which in turn improves their problem-solving skills (Dalgıç, 2011); Leithwood and Steinback (1993) stated that reflective thinking improves school administrators’ problem-solving skills. It is expected that there will be reflection on a group basis among all members in the organization, which leads to an increase in communication between the allies (Vince and Salem, 2004). The person who is expected to pave the way for this is the administrator of that institution and their communication skills.

Unbiased, objective, fair, problem-solving, constructive, strong in human relations and communication, open-minded, constantly, and purposefully thinking, and accessible are expected competencies that a reflective-thinking academic administrator should have. The specified characteristics indicate the characteristics of both the reflective thinker and the administrator. According to Dewey (1933), reflective thinking skill includes thinking open-mindedly on great deal of new ideas, planning the consequences and effects of a particular situation by thinking multidimensionally, and questioning oneself and their actions sincerely in every situation. In this respect, reflective individuals show the characteristics of an administrator who has a critical point of view, is open to development and is responsible for their own learning (York-Barr et al., 2006). The contributions of reflective thinking to academic administrators were determined as improving communication, decision-making and coordination skills the most. In addition, reflective thinking also improves administrators’ organizational and planning and evaluation skills. Considering the characteristics of reflective individuals, it can be said that administrators who can think reflectively can communicate effectively with other members of the organization by having these characteristics, take on the task of making decisions with their leadership skills, take responsibility in the functioning of the organization, and can organize, plan, and evaluate. Reflective school administrators are open to communication with others, so they do not stop oneself as an obstacle to change and development by closing oneself to people inside and outside the institution (Dana, 2009).

When the metaphors developed for reflective-thinking academic administrators are examined, it is revealed that profession and community sub-theme is related to military service, football coach and conductor; nature sub-theme is related to thorn, ramp, mountain climbing; space sub-theme is related to place of worship and pyramid; object sub-theme is related to balloon and flag metaphors. When the metaphors for professions are examined, it can be said that academic administrators think that leadership is the basis of their duties. The military analogy they make for reflective academic administrators emphasizes the rank distribution and hierarchy in the military. A football coach and a conductor are also in the position of direct guides and administrators in their profession. Metaphors for nature are explained with metaphors that give the perception that management is difficult. If the thorn is accepted as the difficulty of the rose, it is desired to state that administration is a beautiful status, but as with every good thing, administration also has difficulties. The ramp is considered as a difficulty encountered on the road, and climbing the mountain is another metaphor chosen to emphasize that being an administrator is a difficult task.

Space metaphors focus on the need for managers to embrace everyone. In this respect, the metaphor of the place of worship emphasizes that reflective-minded administrators should listen to everyone’s problems and support them. Finally, object metaphors reveal that administration is a temporary period. For example, the metaphor of the flag was used to explain the necessity of the administrators to hand over the flags in their hands to another administrator when their term of office ends, as in a relay race. The metaphors that have been created emphasize the need for reflective-thinking administrators to be administrators and to have high communication skills and moreover show that reflective-thinking administrators are aware of the fact that they are doing a beautiful but difficult and temporary task.

Based on the results obtained from the study, the following recommendations can be made:

1. It is recommended that academic administrators be informed about practices that will improve their reflective thinking skills so that they can evaluate oneself and be aware of their strengths and weaknesses.
2. In addition to the individual evaluation of academic administrators’ own competencies, it is recommended that they should be open to the evaluations of fellow academicians and students and include practices that will support the questioning of administration within the institution.
3. It is recommended that academic administrators take an active part in the teaching-learning process as much as possible, even though their administrative work is intense, as it will make it easier for them to see the implementation of the decisions taken as administration from a position outside the administration. In this respect, it is recommended that administrators be in constant communication with the fellow academicians and students and try to be in the same environment.
4. Conducting this study, based on the opinions of deans and vice deans working in different faculties of a public university, on administrators at different education levels will create the opportunity to compare the reflective thinking skills of school administrators at different levels. In addition, it can be suggested that a similar study be conducted based on the opinions of the presidents of the universities. It is also recommended to examine the reflective thinking skills of academic administrators based on the opinions of academics and university students in the institution. The difference between how administrators see oneself and how they are viewed by members of the organization can be explored.
5. The study was based on the assumption that academic administrators working in a university conveyed their experiences and feelings honestly and sincerely, and the study was limited to the views of 12 academic administrators.

## Data Availability Statement

The original contributions presented in this study are included in the article/supplementary material, further inquiries can be directed to the corresponding author.

## Ethics Statement

The studies involving human participants were reviewed and approved by Akdeniz University Rectorate Social and Human Sciences Scientific Research and Publication Ethics Committee. Written informed consent for participation was not required for this study in accordance with the national legislation and the institutional requirements.

## Author Contributions

GK and MD design of the study and wrote sections of the manuscript. EY and MÖ organized the database and performed the qualitative analysis. GK, MD, and MÖ contributed to the manuscript revision. All authors read and approved the submitted version.

## Conflict of Interest

The authors declare that the research was conducted in the absence of any commercial or financial relationships that could be construed as a potential conflict of interest.

## Publisher’s Note

All claims expressed in this article are solely those of the authors and do not necessarily represent those of their affiliated organizations, or those of the publisher, the editors and the reviewers. Any product that may be evaluated in this article, or claim that may be made by its manufacturer, is not guaranteed or endorsed by the publisher.
